# Fusion of Infrared Thermal Image and Visible Image for 3D Thermal Model Reconstruction Using Smartphone Sensors

**DOI:** 10.3390/s18072003

**Published:** 2018-06-22

**Authors:** Ming-Der Yang, Tung-Ching Su, Hung-Yu Lin

**Affiliations:** 1 Department of Civil Engineering, National Chung Hsing University, 145 Xingda Rd., South Dist., Taichung 402, Taiwan; mdyang@dragon.nchu.edu.tw (M.-D.Y.); s940592@gmail.com (H.-Y.L.); 2 Innovation and Development Center of Sustainable Agriculture, National Chung Hsing University, 145 Xingda Rd., South Dist., Taichung 402, Taiwan; 3 Department of Civil Engineering and Engineering Management, National Quemoy University, 1 Da Xue Rd., Kinmen 892, Taiwan

**Keywords:** 3D thermal model, image fusion, smart phone, thermal IR

## Abstract

Thermal infrared imagery provides temperature information on target objects, and has been widely applied in non-destructive testing. However, thermal infrared imagery is not always able to display detailed textures of inspected objects, which hampers the understanding of geometric entities consisting of temperature information. Although some commercial software has been developed for 3D thermal model displays, the software requires the use of expensive specific thermal infrared sensors. This study proposes a cost-effective method for 3D thermal model reconstruction based on image-based modeling. Two smart phones and a low-cost thermal infrared camera are employed to acquire visible images and thermal images, respectively, that are fused for 3D thermal model reconstruction. The experiment results demonstrate that the proposed method is able to effectively reconstruct a 3D thermal model which extremely approximates its corresponding entity. The total computational time for the 3D thermal model reconstruction is intensive while generating dense points required for the creation of a geometric entity. Future work will improve the efficiency of the proposed method in order to expand its potential applications to in-time monitoring.

## 1. Introduction

Currently, three-dimensional (3D) mapping techniques fall into two categories: active or passive mapping techniques. Active mapping techniques, such as laser scanning, directly and quickly acquire an enormous point cloud dataset with high spatial precision. Coupled with visible images, a 3D model with true color information can then be reconstructed. Passive mapping techniques, such as image-based modeling (IBM) [1,2], use image datasets with multiple fields of view (FOV) to reconstruct 3D models, so these techniques usually require certain imaging conditions, including high spatial resolution, a high percentage of imaging lap, accurate camera calibration, and precise camera positioning [3].

Laser scanning can directly provide an enormous point cloud dataset to reconstruct precise 3D models, but requires a high degree of training and is very costly. A light detection and ranging (LiDAR) instrument coupled with a thermal camera has also been applied to 3D thermal model reconstruction [4]. Despite the spatial data acquired by LiDAR instruments having high spatial precision, a LiDAR-based 3D thermal model reconstruction method is still impractical due to its prohibitively high instrument cost. Unlike laser scanning and LiDAR, IBM adopts an indirect method to derive a dataset of transfer parameters, as well as equations for 3D model reconstruction. Furthermore, IBM needs no knowledge about the interior orientation elements of cameras before 3D model reconstruction, and is a low-cost spatial information acquisition technique. With IBM, an effective extraction of conjugate features from adjacent images is necessary. Lowe (2004) [5] indicated that scale-invariant feature transform (SIFT) has invariance to image rotation and scale, and is robust across a substantial range of affine distortion, the addition of noise and change in illumination; thus, it would be useful in key point feature selection. Based on the conjugate features, structure from motion (SFM) can derive the interior orientation elements of a camera in order to reconstruct the 3D model without preprocessing camera calibration [6]. However, the procedure from SIFT to SFM usually requires a central processing unit (CPU) with powerful computation efficiency. In order to address this issue, Wu (2013) [7] ran bundler reconstructions on a PC with an Intel Xenon 5680 3.33 Ghz CPU (24 cores), 12GB RAM, and an nVidia GTX 480 Graphics Processing Unit (GPU). Jancosek and Pajdla (2014) [8] noted that SFM and multiview-stereo (MVS) have become the most popular methods for obtaining input points with visibility information in 3D model reconstruction.

A thermal camera can detect the spectral radiances of objects under a certain temperature, based on the blackbody radiation theory. The detected spectral radiances are usually displayed as digital numbers (DN) obtained by a conversion of radiation level to electric signal. Thus, a thermal image usually consists of numerous DN values, and records temperature information in the two dimensions of rows and columns. Temperature information can be directly extracted from the thermal image, but geometric information, such as edges, feature points and others, cannot; this hampers the understanding of geometric entities consisting of temperature information. Therefore, fusion of visible images with thermal images is helpful for 3D thermal model reconstruction.

Recently, integrations of multisource remote sensing data have been widely applied to environmental monitoring [9,10,11], building inspection [12,13,14,15], heritage preservation [16,17], and non-destructive testing [17,18,19,20,21,22,23]. A 3D thermal model cannot be successfully reconstructed without considering the geometric information in thermal images, thus limiting the above applications. IBM is a computer vision technique for effectively integrating multisource image data into a unique 3D model. Lagüela et al., 2012 [24] used image fusion and image matching techniques to combine thermographic and metric information to obtain 3D thermal models. Several studies also presented the fusion systems for multiple sensors—including 3D laser scanner, RGB camera, and thermal camera—to generate 3D thermal models [17,25,26,27,28]. Therefore, image fusion is an important pre-processing for multiple sensors-based 3D thermal model reconstruction.

With the popularization of smart mobile devices, carried nonmetric sensors have been widely applied in the collection of spatial data due to their convenience, low-cost, and availability [29,30,31]. This study aims to reconstruct 3D thermal models by fusing visible images with thermal images, in which geometric and corresponding temperature information are involved, for the scenes of different scales. The reconstructed 3D thermal models will better display stereo-temperature information compared to conventional thermal images. Building envelop inspection of green buildings, for instance, used 3D thermal models to test the insulation of energy [32,33,34] and to assess the failure of external wall tiles [35]. In this paper, a cost-effective sensor on smart phones for 3D thermal model reconstruction based on IBM is proposed, and the performances, including model quality and computational time, of the 3D thermal model reconstruction are discussed.

## 2. Experimental Equipment

This study used two smart phones (iPhone SE) and a low-cost thermal camera (FLIR ONE for iOS) to acquire visible images and thermal IR images, respectively. The iPhone SE specifications are 12 million pixels (3000 pixels in the row direction and 4000 pixels in the column direction) and a focal length of 4.15 mm. The FLIR ONE specifications are 76,800 pixels (240 pixels in the row direction and 320 pixels in the column direction), a focal length of 3 mm, and a range of detected temperature between −20 and 120 °C (±3 °C or ±5%). The FLIR ONE also carries a visible light lens with the same number of pixels and focal length as the thermal one.

**Figure 1:**
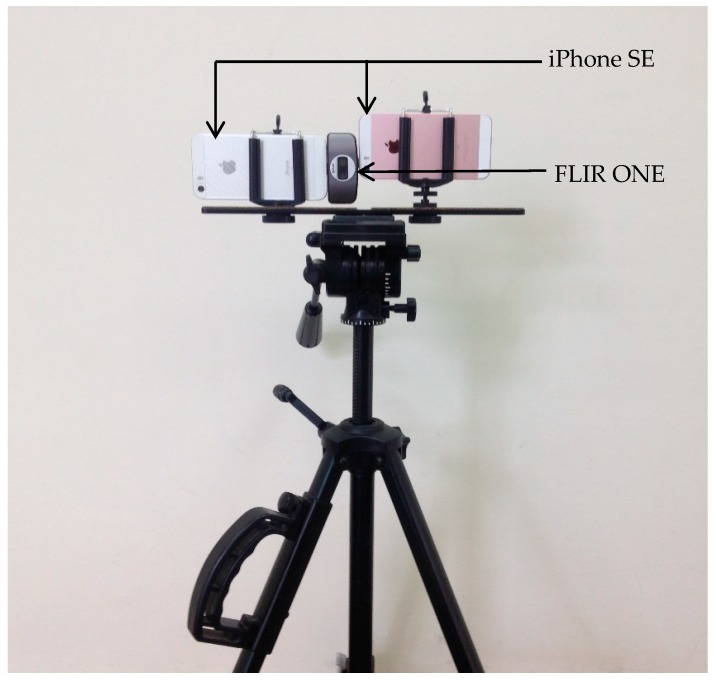
Arrangement of experimental equipment.

Figure 1 shows the arrangement of the experimental sensors. Two smart phones were used to acquire stereopairs of visible images with high spatial resolution. The FLIR ONE was arranged between the two smart phones to acquire the thermal infrared (IR) images. Thus, an alignment arrangement of the three sensors on a theodolite ensures that the exterior orientation elements of the sensors are known. Moreover, the shifts among the lenses are fixed, which facilitates the geometric calibration for the acquired images.

## 3. Methodology

**Figure 2:**
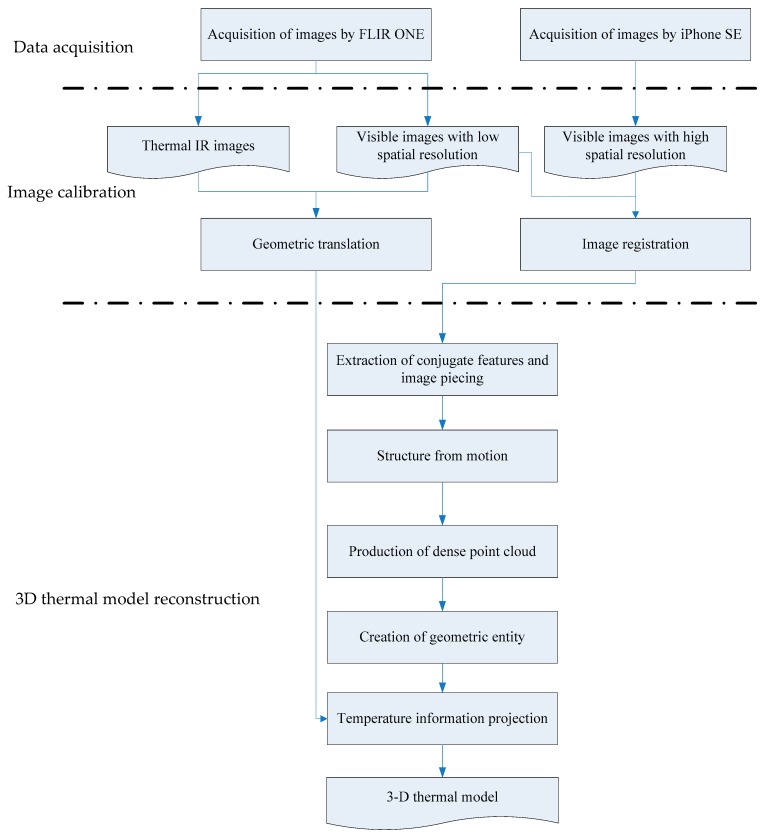
Scheme of proposed method for 3D thermal model reconstruction.

Figure 2 shows the proposed 3D thermal model reconstruction method. The first stage is to acquire the visible and thermal IR images. Next, the acquired images are calibrated by geometric translation and image registration using normalized cross correlation (NCC) [36]. The geometric translation addresses the shift between the visible and thermal IR lenses on the FLIR ONE. The image registration matches the visible images of the FLIR ONE with those of the iPhone SE. In the final stage, the visible images of the iPhone SE are used to reconstruct a 3D model by the conventional IBM technique, and the geometric translated thermal IR images are textured onto the reconstructed 3D model to produce a 3D thermal model.

### 3.1. Image Calibration

Generally, a baseline between two photographs with stereoscopic viewing must be fixed so that the shift between the two camera stations is known a priori in order to produce a good geometric calibration. In addition to the known baseline, the correct interior orientation elements of the experimental equipment are also required. However, acquiring the correct interior orientation elements from a manufacturer calibration is extremely difficult due to the limited budget. In this research, several algorithms, such as NCC and three-step search (TSS) [37]—encoded in the C++ and OpenCV programming languages—were tested for image calibration.

#### 3.1.1. Normalization of Sensed Temperature

The FLIR ONE offers relative sensed scenes rather than absolute temperature information, so that a certain DN value among different thermal IR images can stand for different sensed temperatures. In this paper, a normalization technique is presented to appropriately adjust the obtained DN values in order to achieve consistency in sensed temperature. The normalization equations are as follows

(1) $${\mathrm{DN}}_{\min}=\left[\left(255-0\right)/\left(T_{\max}-T_{\min}\right)\right]\times \left(T_{L}-10.0\right)$$

(2) $${\mathrm{DN}}_{\max}=\left[\left(255-0\right)/\left(T_{\max}-T_{\min}\right)\right]\times \left(T_{H}-10.0\right)$$

(3) $$\frac{{\mathrm{DN}}_{a}-{\mathrm{DN}}_{\min}}{{\mathrm{DN}}_{b}-0}=\frac{{\mathrm{DN}}_{a}-{\mathrm{DN}}_{\max}}{{\mathrm{DN}}_{b}-255}$$

(4) $${\mathrm{DN}}_{a}=\frac{{\mathrm{DN}}_{b}\left({\mathrm{DN}}_{\max}-{\mathrm{DN}}_{\min}\right)+255{\mathrm{DN}}_{\min}}{255}$$

where DN_max_ and DN_min_ are the normalized DN values of the highest and lowest sensed temperatures, respectively. DN_b_ and DN_a_ express the DN values before and after the normalization, respectively. T_H_ and T_L_ are the highest and lowest sensed temperatures before the normalization, respectively, and T_max_ and T_min_ are the highest and lowest sensed temperatures after the normalization, respectively. As the thermal IR images are 8-bit images, the sensed temperature information can be expressed as the DN values between 0 (corresponding to T_min_) and 255 (corresponding to T_max_).

#### 3.1.2. Geometric Translation

**Figure 3:**
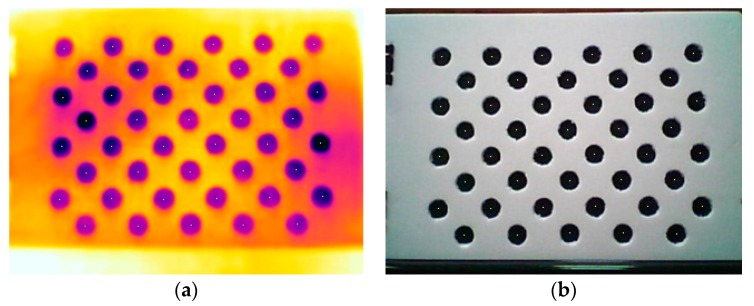
Calibration template for geometric translation: (**a**) false color thermal IR image; (**b**) gray scale visible image.

Due to the shift between the thermal IR and visible lenses on the FLIR ONE, an image mapping result could have the phenomenon of relief displacement. In other words, relief displacement is zero for one lens at the nadir point but not zero for the other one at the off-nadir point [38]. To ensure that the percentage of end lap is fixed, a unity object distance among the acquired images is also needed. Consequently, the amount of relief displacement can be calculated prior to the 3D model reconstruction. A calibration template, shown in Figure 3, is employed to acquire 10 pairs of thermal IR and visible images for the geometric translation, where firstly the centers of the circles on the calibration template are detected by OpenCV and secondly a root mean square error (RMSE) of the coordinates of the detected circular centers between the thermal IR and visible images is calculated. According to RMSE, the circular centers between the thermal IR and visible images can be translated to the consistent positions.

#### 3.1.3. Image Registration

Image registration matches the visible images of the FLIR ONE with those of the iPhone SEs. Because the visible images of the FLIR ONE and iPhone SE differ in size, an image resampling process is necessary before image registration. Thus, the iPhone SE images need to be down-sampled so to have the same spatial resolution as those captured by the FLIR ONE. The percentage of down-sampling affects the image registration performance. The actual focal lengths of the FLIR ONE and iPhone SE are 3 mm (f_1_) and 4.15 mm (f_2_), respectively, and have a relationship with object distance (d) and size of object (l) as

(5) $$\frac{f_{1}}{l_{1}}=\frac{f_{2}}{l_{2}}=\frac{d}{l}$$

(6) $$\{\begin{array}{c} l_{1}\;=\;P_{1}\times S_{1}\\ l_{2}\;=\;P_{2}\times S_{2} \end{array}$$

where P_1_ and P_2_, and S_1_ and S_2_ represent the pixel numbers, and the sizes of charge-coupled devices (CCD) of the FLIR ONE and iPhone SE, respectively. Thus, l_1_ and l_2_ express the sizes of CCD arrays of the FLIR ONE and iPhone SE, respectively. According to Equation (5), if S_1_ and S_2_ are known, a certain percentage of down-sampling exists between P_1_ and P_2_. In order to obtain an optimal percentage of down-sampling, the percentages of down-sampling from 8% to 50% were tested. The optimal percentage of down-sampling leads to an image registration result with the least RMSE.

**Figure 4:**
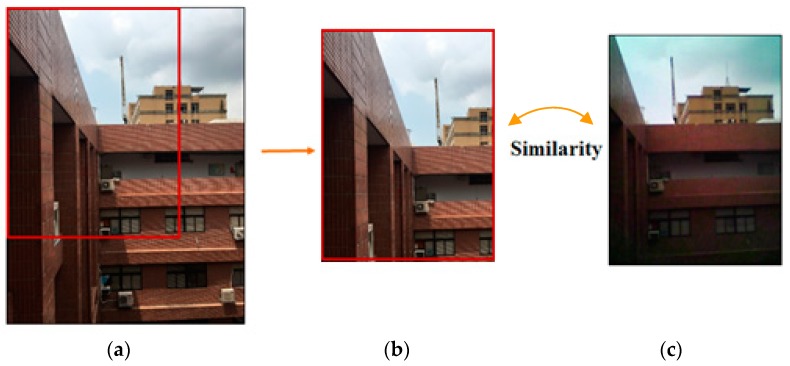
Illustration of image registration using NCC: (**a**) original image of iPhone SE. Down-sampling visible iPhone SE image. Spatial resolution: 4032 × 3204 ×*k*%; (**b**) template image. Spatial resolution: 320 × 240; (**c**) registered image. Visible FLIR ONE image. Spatial resolution: 320 × 240.

NCC is a common image registration technique as

(7) $$\mathrm{NCC}\;\mathrm{Index}=\frac{1}{n}\underset{x,y}{\sum }\frac{\left(f\left(x,y\right)-\bar{f}\right)\left(t\left(x,y\right)-\bar{t}\right)}{\sigma_{f}\sigma_{t}}$$

where f(x,y) and t(x,y) represent the DN values of the registered image and the template image, respectively. $\bar{f}$ and $\bar{t}$ and $\sigma_{f}$ and $\sigma_{t}$ are the means and the standard deviations of DN values of the registered image and the template image, respectively. x and y are the numbers of pixels in the row and column directions, respectively. The values of x and y are given as 320 and 240, respectively, because of the 320 × 240 FLIR ONE thermal IR images (as registered images). Figure 4 is an illustration of image registration using NCC. The k% is a percentage of down-sampling from the original image of iPhone SE. The image region within the red frame is regarded as a template image with 320 × 240 pixels subset from the k% down-sampling iPhone SE image and from the upper left to the lower right to calculate the NCC indices of all template images. The NCC index ranges between −1 and 1, and means a high similarity between the registered image and template image while approaching 1.

**Figure 5:**
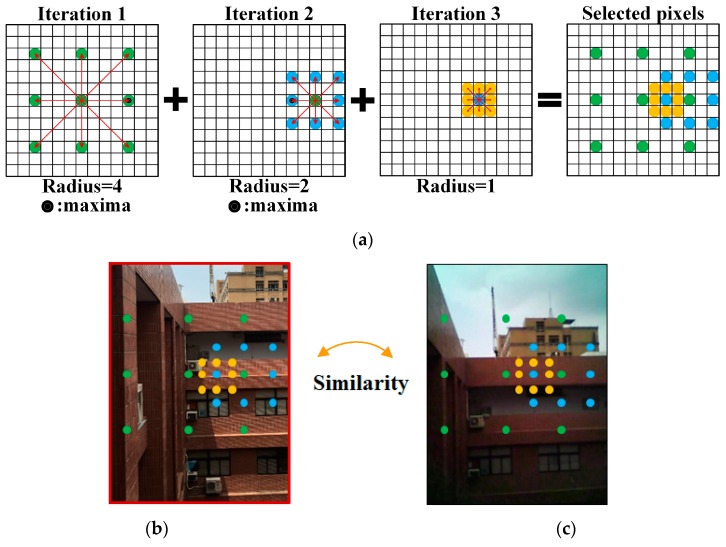
Illustration of TSS operation: (**a**) pixels selected to be examined in similarity; (**b**) selected pixels on template image; (**c**) selected pixels on registered image.

However, the computation complexity of NCC depends on the sizes of the processed images, and the process is usually time-consuming. Li et al., 1994 [36] noted that TSS is an efficient algorithm in processing an enormous imagery dataset, and thus could be frequently applied to film detection. In this research, TSS was adopted to facilitate NCC in the image registration. In this research, TSS starts the NCC-based search from the central pixel of a visual image of FLIR ONE with the search radius S pixels. Thus, TSS searches eight pixels around the central one with a search radius of S pixels. Of these eight searched pixels, the pixel with the least DN difference compared to the registered image is selected to be a new search origin [39]. Then, the search radius is reduced by half, i.e., 0.5S pixels, and repeats a similar search for several iterations until the search radius is equal to 1 pixel. Finally, TSS assists NCC in efficiently finding a template image with the best match with the registered image. The computational time and number of search pixels are significantly reduced. A full search of NCC requires x × y pixels whereas TSS with *n* iterations only needs 8*n* + 1 (or 9 + (*n* − 1) × 8) pixels [40]. Figure 5 shows the TSS process setting S = 4 pixels for example, in which the green, blue, and orange pixels are the selected pixels to be examined in similarity in the first, second, and third iterations, respectively.

### 3.2. 3D Thermal Model Reconstruction

A 3D thermal model reconstruction procedure includes extraction and matching of conjugate features, structure from motion, production of dense point cloud, creation of geometric entity, and temperature information texturing. The above steps are described below. Especially, extraction and matching of conjugate features, and structure from motion were implemented by an open GUI application, i.e., VisualSFM [41]. An open resource, i.e., Open MVS [42], was employed in production of dense point cloud, and creation of geometric entity.

#### 3.2.1. Extraction of Conjugate Features and Image Piecing

The conjugate feature extraction performance affects the creation of a geometric entity. Scale-invariant feature transform (SIFT), a popular computer vision technique, offers image matching with invariance of scale, rotation, and brightness [5]; therefore, SIFT can extract appropriate and sufficient conjugate features from an enormous imagery dataset, and is useful in image piecing under any imaging condition.

The first step of conjugate feature detection is to identify locations and scales that can be repeatably assigned under differing views of the same object. Location detection is invariant to scale change of an image that can be accomplished by searching for stable features across all possible scales, using a scale-space kernel of Gaussian function

(8) $$G\left(x,y,\;\sigma \right)=\frac{1}{2\pi \sigma^{2}}e^{\frac{-\left(x^{2}+\;y^{2}\right)}{2\sigma^{2}}}$$

Therefore, the scale space of the image is defined as a function, *L*(*x*, *y*, σ)

(9) L(x, y, σ)=G(x,y, σ)∗ I(x, y),

where *I*(*x*, *y*): the image, *: the convolution operation in *x* and *y*.

Next, a difference-of-Gaussian function convolved with the image was used to efficiently detect stable conjugate feature locations in the scale space. The difference, *D*(*x*, *y*, σ), is expressed as

(10) D(x, y, σ)=L(x,y, kσ)− L(x,y, σ)

Lowe (2004) [5] used a Taylor expansion (up to the quadratic terms) of the scale-space function, *D*(*x*, *y*, σ), shifted so that the origin is at the feature point x

(11) $$D\left(x\right)=D+\;\frac{\partial D^{T}}{\partial x}+0.5x^{T}\frac{\partial^{2}D}{\partial x^{2}}x$$

where *D* is a detected conjugate feature by the difference, x = (*x*, *y*, σ)^T^. The location of the extremum, $\hat{x}$, is determined by taking the derivative of Equation (11) with respect to x and $\hat{x}$ is

(12) $$\hat{x}=\;\frac{\partial^{2}D^{-1}}{\partial x^{2}}\frac{\partial D}{\partial x}=0$$

If the offset $\hat{x}$ is larger than 0.5 (the image pixel values assumed in the range [0,1]) in any dimension, it means that the $\hat{x}$ lies closer to a different feature point. In this case, the feature point is changed and an interpolation performed instead of the point. The final offset is added to the location of the detected conjugate feature *D* to obtain the interpolated estimation for the location of the $\hat{x}$

(13) $$D\left(\hat{x}\right)=D+0.5\frac{\partial D^{T}}{\partial x}\hat{x}$$

If a value of $\left|D\left(\hat{x}\right)\right|$ is less than 0.03, the detected feature *D* is discarded.

#### 3.2.2. Structure from Motion (SFM)

Based on the conjugate features extracted by SIFT, the camera stations can be determined according to the geometries of the epipolar planes; thus, a geometric relationship can be established to reconstruct the 3D model for an imaged scene. Assuming existing one pair of images *I* and *J*; F(I) is defined as set of conjugate features in image *I*, and conjugate feature in the set is defined as *f_i_* ∈ F(I) and the correspondence of image *J* is defined as *f_j_*∈ F(J). Eventually, the Euclidean distance (the shortest two conjugate features between two images) is searched and their correspondence is established. This study define image *J* corresponds to the closest to the conjugate feature on the image *I* as $f_{nn}$ ∈ F( J) as [43]

(14) $$f_{nn}=argmin_{f^{\prime }\in F\left(J\right)}{\left\|f_{i}-f_{j}\right\|}_{2}$$

The epipolar geometry of an image pair can be expressed in a 3 × 3 rank-2 matrix, so-called fundamental matrix ***F***, which describes the relative positions and orientations of the two sensors as well as internal sensor settings such as zoom. Each pair of corresponding points (x, y) → (x’, y’) in two corresponding images must satisfy the epipolar constraint [44]

(15) $$x^{\prime }Fx^{T}=0$$

(16) $$F\sim K^{-1T}\left[T\right]\times RK^{\prime -1}$$

where x = [*xy* 1], x’ = [*x’y’* 1]. *R* is the rotation matrix, and *K* is the intrinsic sensor matrix as

(17) $$K=\left[\begin{array}{ccc} k_{11} & k_{12} & k_{13}\\ 0 & k_{22} & k_{23}\\ 0 & 0 & 1 \end{array}\right]$$

In order to optimize the geometric relationship, a bundle adjustment—based on the derived triangular points, which involve reconstructing 3D coordinates of the corresponding features by detecting the motion tracking orientation of features, and camera stations—is employed in this study to repeatedly implement the collinearly forward or backward intersections until the residual errors are converged [45]. Eventually, all interior parameters can be modified through bundle adjustment [2].

In the bundle adjustment, this paper considered that a sensor exterior matrix includes the rotation matrix *R* and a translation vector *T*. To reduce the parameters, an incremental rotation $R\left(\theta ,\hat{n}\right)$ is defined as [43]

(18) $$R\left(\theta ,\hat{n}\right)=I+sin\theta {\left[\hat{n}\right]}_{\times }+\left(1-cos\theta \right){\left[\hat{n}\right]}_{\times }^{2},\;\omega =\theta \hat{n},\;{\left[\hat{n}\right]}_{\times }=\left[\begin{array}{ccc} 0 & -{\hat{n}}_{z} & {\hat{n}}_{y}\\ {\hat{n}}_{z} & 0 & -{\hat{n}}_{z}\\ -{\hat{n}}_{y} & {\hat{n}}_{z} & 0 \end{array}\right]$$

where θ is an angle of rotation respect to a three-vector unit axis, $\hat{n}$. $R\left(\theta ,\hat{n}\right)$ is pre-multiplied by the initial rotation matrix to compute the current rotation inside the global optimization. $R\left(\theta ,\hat{n}\right)$ is nearly linear in ω for small incremental rotations. A non-linear iterative optimization approach, such as the Gauss–Newton iterative method [2], was adopted to minimize $R\left(\theta ,\hat{n}\right)$. Let θ→θ+δθ and f(θ) be smaller. According to the Taylor series

(19) $$f\left(\theta +\delta \theta \right)\approx f\left(\theta \right)+g^{T}\delta \theta +\frac{1}{2}\delta \theta^{T}H\delta \theta$$

where g is the gradient, and H is the Hessian matrix. Let $\frac{\mathrm{df}}{d\theta }\left(\theta +\delta \theta \right)\approx g+H\delta \theta \approx 0$

(20) $$\delta \theta =-H^{-1}g$$

Equation (20) can be rewritten in term of Jacobian J as Gauss-Newton or normal equations

(21) $$(J^{T}\mathrm{WJ})\delta \theta =-J^{T}W\Delta z$$

Equation (21) was used to evaluate the minimum for well-parameterized bundle problems under an outlier-free least squares [44]. Finally, the features derived by SFM were loaded in a custom coordinate system according to the relative point positions.

#### 3.2.3. Production of Dense Point Cloud

After the bundle adjustment, the optimized triangular points are obtained and assembled as a point cloud. The preliminary point cloud is fairly sparse, so this study introduces a nearest-neighbor field (NNF) algorithm [46] for the production of a dense point cloud. In NNF, the first step is to build a few initial kernels based on several triangular points. Given triangular point **a** in image ***A*** and its corresponding triangular point **b** in image ***B***, the value of *f*(*a*) as offset is simply **b** − **a**. The following step is to randomly move the kernels and simultaneously execute an imagery consistency test along with the moving kernels. In order to improve the offsets of *f*: ***A***→**R**^2^, a sequence of candidate offsets at an exponentially decreasing distance from *f* was tested [46]

(22) $$u_{i}=f+w\alpha^{i}R_{i}$$

where **R***_i_* is a uniform random in [−1, 1] × [−1, 1], *w* is a large maximum search radius, and α is a fixed ratio between the kernels. Equation (22) was examined for *i* = 0, 1, 2, … until the current search radius $w\alpha^{i}$ is below 1 pixel. In this paper, *w* is the maximum image dimension, and α = 0.5, except where noted. Note the kernels must be clamped to the bounds of ***B***. The final step is to produce new points within the kernels, which can pass the imagery consistency test, and to repeat the second through final steps to improve the density of a point cloud.

#### 3.2.4. Creation of Geometric Entity

Visual hull presented by Laurentini (1994) [47] is used to create the geometric 3D model. Visual hull assumes the foreground object in an image can be separated from the background, thus the original image can be segmented into a foreground/background binary image, called a ‘silhouette image’. The foreground mask, known as a silhouette, is the 2D projection of the corresponding 3D foreground object. Along with the camera viewing parameters, the silhouette defines a back-projected generalized cone that contains the actual object, so-called a silhouette cone. Thus, the 2D projection is obtained by an intersection of the intersected silhouette cones and the emitted rays from sensed object to sensor.

In this research, Visual hull was adopted to derive the point cloud, i.e., foreground point cloud, of a sensed object from the dense point cloud dataset. Coupled with the method of Delaunay triangulation [48], a mesh of the sensed object can be built based on the foreground point cloud to create the geometric entity.

#### 3.2.5. Temperature Information Texturing

Using the image calibration described in Section 3.1, the relationship between the images acquired by the FLIR ONE and iPhone SEs is established. Consequently, the thermal IR images can be directly textured onto the surface of the created 3D model by the collinearity theory. The collinearity condition equations for temperature information texturing are expressed as

(23) $$\{\begin{array}{c} x=x_{0}-f_{1}\left[\frac{m_{11}\left(X-X_{L}\right)+m_{12}\left(Y-Y_{L}\right)+m_{13}\left(Z-Z_{L}\right)}{m_{31}\left(X-X_{L}\right)+m_{32}\left(Y-Y_{L}\right)+m_{33}\left(Z-Z_{L}\right)}\right]\\ y=y_{0}-f_{1}\left[\frac{m_{21}\left(X-X_{L}\right)+m_{22}\left(Y-Y_{L}\right)+m_{23}\left(Z-Z_{L}\right)}{m_{31}\left(X-X_{L}\right)+m_{32}\left(Y-Y_{L}\right)+m_{33}\left(Z-Z_{L}\right)}\right] \end{array},$$

where (*x*, *y*) is the space coordinate of an object point in a thermal IR image plane; (*x*_0_, *y*_0_) is the principal point of a thermal IR image; *f*_1_ is the focal length of the FLIR ONE; (*X_L_*, *Y_L_*, *Z_L_*) is the coordinate, with respect to the entity coordinate system *XYZ*, of exposure station of a thermal IR image; the *m*’s are functions of the rotation angles along the *XYZ* axes of the FLIR ONE.

## 4. Results and Discussion

### 4.1. Geometric Translation of Double Lenses of FLIR ONE

Based on the 10 pairs of thermal IR and visible images of the calibration template and an object distance of 2 m, the geometric translations in the column and row directions were calculated in Table 1. The average geometric translations in the column and row directions are 4.771 and −2.321 pixels, respectively. According to the average geometric translations, the phenomenon of relief displacement is effectively removed from the FLIR ONE thermal IR and visible images.

### 4.2. Image Registration of Visible Images of FLIR ONE and iPhone SE Images

Down-sampling percentages from 8% to 50% were tested for the registration of the visible FLIR ONE and iPhone SE images. It is worth noting that the spatial resolution of iPhone SE images is lower than that of FLIR ONE images, if the down-sampling percentage is lower than 8%. Table 2 lists the RMSE values of the image registration for different down-sampling percentages, and shows that 9% down-sampling results in the best image registration. Consequently, this paper further tested the 9% down-sampling with the centesimal percentages to derive the RMSE values of the image registration (see Table 3). The experiment results indicate the best down-sampling percentage of 9.24%.

**Figure 6:**
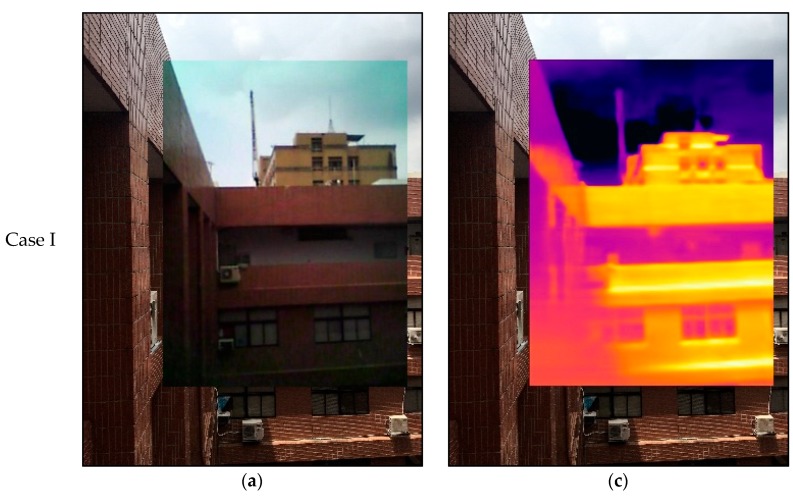

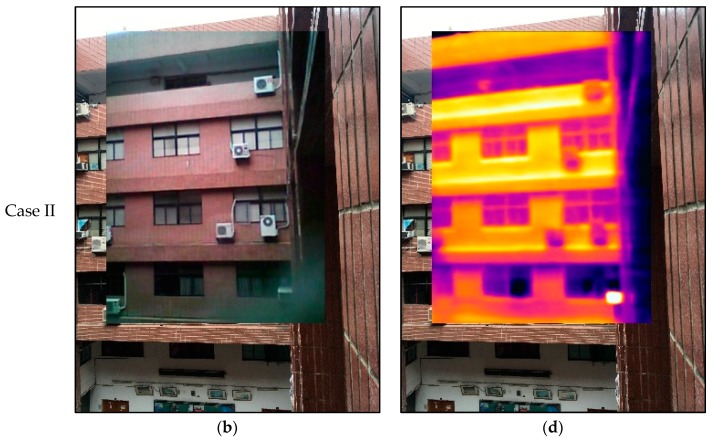
Image registration of exterior wall of departmental building: (**a**,**b**) Visible image of FLIR ONE registered for that of iPhone SE; (**c**,**d**) Thermal IR image of FLIR ONE registered for visible image of iPhone SE.

Three scenes of the exterior wall of the departmental building of Civil Engineering and Environmental Engineering at National Chung Hsing University (NCHU) (Taichung, Taiwan), Taichung, Taiwan were imaged and provided for an image registration test, as shown in Figure 6. Table 4 compares the performances of the image registration by NCC with and without assistance from TSS. In addition, the computational times required by the Intel(R) Core(TM) i7-3610QM CPU are listed in Table 4. NCC without the assistance of TSS needs about 321 s to derive the NCC indices of approximately between 0.95 and 0.96. Nevertheless, it is demonstrated that TSS can effectively assist NCC in improving the NCC indices from 0.95 to 0.99, and simultaneously reduce computational time significantly.

### 4.3. 3D Thermal Model Reconstruction

**Figure 7:**
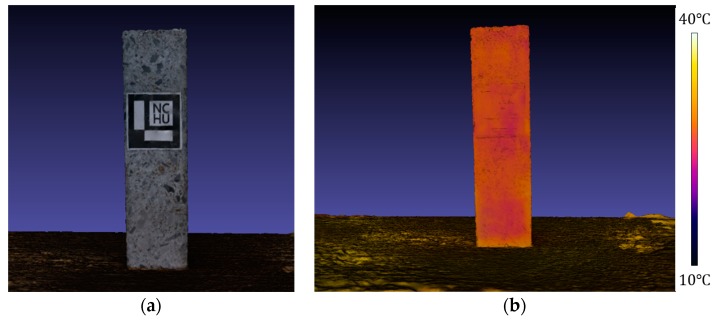
Model reconstruction of small scale scene: (**a**) 3D model of concrete sample; (**b**) 3D thermal model of concrete sample.

This section discusses the 3D thermal model reconstruction performance of the proposed method for small, medium, and large-scale scenes and the computational time for each. A concrete sample for strength testing was selected as the small-scale 3D thermal model reconstruction scene (see Figure 7). The volume of the concrete sample was found to be 3662.5 and 3404.7 cm^3^ by the drainage method and the proposed method, respectively. Thus, the volume error of the 3D thermal model reconstruction is 7.04%. Additionally, conventional IBM was also used to determine the volume of the concrete sample. The determined volume and volume error are 3517.7 cm^3^ and 3.95%, respectively. In terms of 3D model reconstruction accuracy, the proposed method is slightly inferior to the conventional IBM, but the 3D model reconstructed by the proposed method contains temperature information. Consequently, the proposed method is demonstrated as an approximately cost-effective 3D thermal model reconstruction method, but the method still needs further development to improve volume accuracy of the 3D thermal model.

**Figure 8:**
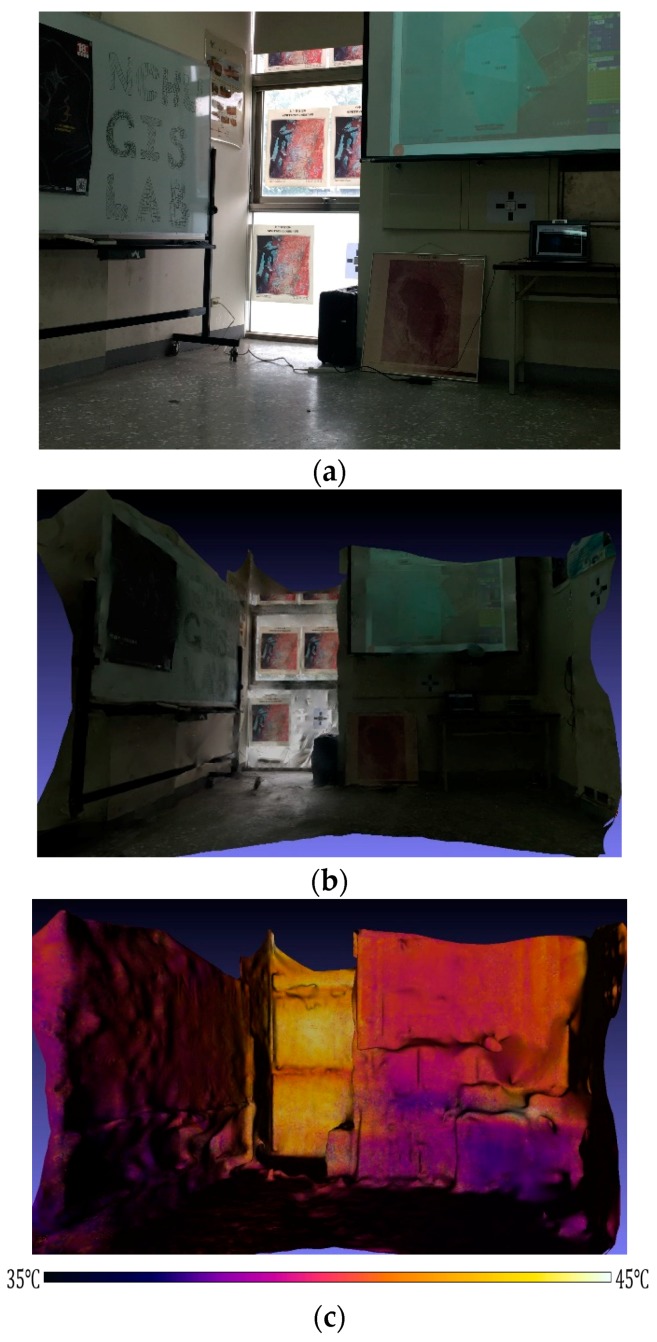
Model reconstruction of medial scale scene: (**a**) visible image of classroom scene; (**b**) 3D model of classroom scene; (**c**) 3D thermal model of classroom scene.

A classroom in the departmental building was selected as the medium-scale 3D thermal model reconstruction scene (see Figure 8). The 3D thermal model shows that the detected temperature of the classroom is between 35° and 45°, and the precision of the temperature information is ±2°. The temperature information precision was estimated by a comparison of the normalized sensed temperatures of several feature points with their corresponding thermograms in situ. The temperature of the glass curtain wall is over 40°, which is significantly higher than that of the interior of the classroom. If the classroom is intended to be part of a green building policy, the performances of proposed energy efficiency strategies can be presented and compared using this 3D thermal model.

**Figure 9:**
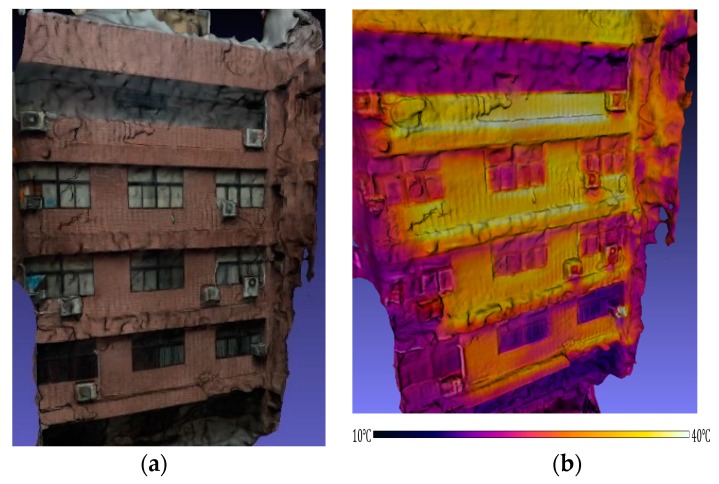
Model reconstruction of large scale scene: (**a**) 3D model of exterior wall of departmental building; (**b**) 3D thermal model of exterior wall of departmental building.

An exterior wall of the departmental building was selected as the large-scale 3D thermal model reconstruction scene (see Figure 9). The temperature of the tile wall is higher than that of the window glass. Moreover, the upper tile wall compared to the lower one seems to have a higher temperature. Due to the shading of the floors, the corridors have a lower temperature than the tile wall. In the winter of 2016, the departmental building experienced an extreme cold-front, causing several tiles to detach from the exterior wall. The proposed method could, in the future, also provide multi-temporal 3D thermal models for exterior wall tests to pre-empt such deterioration.

In Figure 8 and Figure 9, the sensed scenes, i.e., the classroom and the exterior wall, include the multiple emissivities, which can vary with sensed wavelength, viewing angle, and temperature [49], of the different materials, thus in the future the reconstructed 3D thermal models also can be applied to inversely calculate the emissivity of a certain material if the sensed wavelength and viewing angle are known.

### 4.4. Computational Time

Table 5 lists the number of processed images and the required computational time for each step of the proposed scheme in Figure 2. The larger the scene, the longer the computational time requires; however, the total computational time does not seem to increase with an increase of the number of processed images. Table 5 indicates that the bottleneck step should be “creation of geometric entity” due to its needs of computational time significantly more than other steps.

Table 6 shows the qualities of the 3D models derived by structure from motion, production of dense point cloud, and creation of geometric entity. The production of a dense point cloud could produce a dozen times the number of points than by deriving structure from motion. Despite this, there is no significant relationship between the numbers of the dense points and the silhouette cones. In other words, only a portion of the dense points are useful in the creation of geometric entity, and most of the dense points may be superfluous. Whelan et al., 2015 [50] also indicated that planar segments have a simple shape which can be well described by points on the boundary of the segment. Interior points only add redundancy to the surface representation and complicate the triangulation results. If the appropriate number of dense points required for the creation of a geometric entity can be determined in advance, the computational time of the creation of a geometric entity should be significantly reduced, thus reducing total computational time.

Table 5 and Table 6 are noticed that the medial scale scene has the less number of points but needs the more computational time in creation of geometric entity than the small scale one. According to the implementation of Open MVS, 553 s of 2650 s and 70 s of 31,453 s were taken to derive the foreground point cloud datasets of the concrete sample (small scale scene) and the classroom (medial scale scene), respectively. Due to the more complicated surface textures of the classroom scene than the concrete sample, Open MVS based on the foreground point cloud datasets took much more time to mesh the classroom scene than the concrete sample.

## 5. Conclusions

This paper presents an IBM-based method for 3D thermal model reconstruction; two smart phones and a low-cost thermal camera were employed to acquire visible images and thermal images, respectively, that was fused for constructing 3D thermal models. In the IBM-based method, image calibration, which includes geometric translation and image registration, is an important pre-processing for 3D thermal model reconstruction. It was demonstrated that TSS can effectively assist NCC in reducing the computational time required for image registration, and that the optimal percentage for down-sampling the smartphone image size to that of the thermal camera is 9.24%.

A small scene, i.e., concrete sample, was tested to reconstruct its 3D thermal model, and the obtained volume error of the 3D thermal model is 7.04%. A classroom within the departmental building and an exterior wall of the departmental building were tested to reconstruct their 3D thermal models. The normalized sensed temperatures of several feature points were compared with their corresponding thermograms in situ and show a temperature precision of 2 °C in the 3D thermal models.

According to the required computational time of 3D thermal model reconstruction, the creation of a geometric entity, one of the steps of 3D thermal model reconstruction, is the critical step. Moreover, the production of superfluous points in the point cloud increases the computational time, reducing the efficiency of 3D thermal model reconstruction. At present, it has been demonstrated that the proposed method is approximately cost-effective in 3D thermal model reconstruction. If the appropriate number of dense points required for the creation of a geometric entity can be determined in advance, the computational time of 3D thermal model reconstruction can be significantly reduced. Future work, such as removing redundant points from dense point cloud by the method of Whelan et al., 2015 [50], will continue to improve the method in order to further reduce computational time and to offer better model quality and in-time monitoring. Moreover, a metric scale associated with the detected objects should also be considered in the further development of 3D thermal model reconstruction.

## Figures and Tables

**Table 1:** Calculation results of the geometric translation.

ID of Pair of Images	Geometric Translation in the Column Direction (Pixels)	Geometric Translation in the Row Direction (Pixels)
IMG_L_1	3.579	−1.289
IMG_L_2	4.777	−1.095
IMG_L_3	6.805	−3.532
IMG_L_4	5.789	−3.193
IMG_L_5	3.153	−1.238
IMG_L_6	6.210	−2.764
IMG_L_7	4.446	−2.374
IMG_L_8	4.274	−2.737
IMG_L_9	4.498	−2.684
IMG_L_10	4.177	−2.305
Average	4.771	−2.321

**Table 2:** RMSE of image registration varying with down sampling percentages.

Down Sampling Percentage	RMSE (Pixels)
8%	15.712
9%	3.403
10%	9.560
20%	211.380
30%	427.958
40%	856.391
50%	1064.691

**Table 3:** RMSE of image registration under 9% down sampling with centesimal percentages.

Down Sampling Percentage	RMSE (Pixels)
9.20%	2.818
9.22%	2.738
9.24%	1.846
9.26%	2.041
9.28%	2.564
9.30%	2.629
9.40%	2.831
9.60%	4.230
9.80%	6.712

**Table 4:** NCC indices and computational time of image registration.

Case	NCC		NCC + TSS	
	NCC Index	Computational Time (s)	NCC Index	Computational Time (s)
I	0.955	321.188	0.977	1.676
II	0.957	323.016	0.995	1.584
III	0.963	321.089	0.991	1.721

**Table 5:** Computational time of 3D thermal model reconstruction.

Step	Small Scale Scene		Medial Scale Scene		Large Scale Scene	
	No._(I)_^1^	CT(s) ^2^	No._(I)_^1^	CT(s) ^2^	No._(I)_^1^	CT(s) ^2^
1. Image calibration	81	219	202	508	51	69
2. Extraction of conjugate features and image piecing		165		231		192
3. Structure from motion		486		1686		1091
4. Production of dense point cloud		912		167		2365
5. Creation of geometric entity		2650		31,453		36,323
6. Temperature information projection		131		311		373
Total		4563		34,356		40,413

^1^ Number of images; ^2^ Computational time.

**Table 6:** Qualities of 3D models derived by structure from motion, production of dense point cloud, and creation of geometric entity.

Scene	Step	No. of Points	No. of Silhouette Cones
Small scale	Structure from motion	46,911	-
	Production of dense point cloud	377,785	-
	Creation of geometric entity	-	753,627
Medial scale	Structure from motion	10,515	-
	Production of dense point cloud	270,368	-
	Creation of geometric entity	-	540,022
Large scale	Structure from motion	735,440	-
	Production of dense point cloud	13,587,715	-
	Creation of geometric entity	-	509,281

## References

[1] Oliveira M.M. (2002). Image-based modeling and rendering techniques: A survey. RITA.

[2] Yang M.D., Chao C.F., Lu L.Y., Huang K.S., Chen Y.P. (2013). Image-based 3D scene reconstruction and exploration in augmented reality. Autom. Constr..

[3] Nguyen T.T., Slaughter D.C., Max N., Maloof J.N., Sinha N. (2015). Structured light-based 3D reconstruction system for plants. Sensors.

[4] Yandún Narváez F.J., Salvo del Pedregal J., Prieto P.A., Torres-Torriti M., AuatCheein F.A. (2016). LiDAR and thermal images fusion for ground-based 3D characterisation of fruit trees. Biosyst. Eng..

[5] Lowe D.G. (2004). Distinctive image features from scale-invariant keypoints. Int. J. Comput. Vis..

[6] Yang Z. Fast Template Matching Based on Normalized Cross Correlation with Centroid Bounding. Proceedings of the 2010 International Conference on Measuring Technology and Mechatronics Automation (ICMTMA).

[7] Wu C. Towards Linear-time Incremental Structure from Motion. Proceedings of the 2013 International Conference on 3D Vision (3DV 2013).

[8] Jancosek M., Pajdla T. (2014). Exploiting visibility information in surface reconstruction to preserve weakly supported surfaces. Int. Sch. Res. Not..

[9] Giuliani G., Dao H., De Bono A., Chatenoux B., Allenbach K., De Laborie P., Rodila D., Alexandris N., Peduzzi P. (2017). Live monitoring of earth surface (LiMES): A framework for monitoring environmental changes from earth observations. Remote Sens. Environ..

[10] Manzo C., Mei A., Zampetti E., Bassani C., Paciucci L., Manetti P. (2017). Top-down approach from satellite to terrestrial rover application for environmental monitoring of landfills. Sci. Total Environ..

[11] Gulbe L., Caune V., Korats G. (2017). Urban area thermal monitoring: Liepaja case study using satellite and aerial thermal data. Int. J. Appl. Earth Obs. Geoinf..

[12] Roca D., Lagüela S., Díaz-Vilariño L., Armesto J., Arias P. (2013). Low-cost aerial unit for outdoor inspection of building façades. Autom. Constr..

[13] Natephra W., Motamedi A., Fukuda T., Yabuki N. (2017). Integrating building information modeling and virtual reality development engines for building indoor lighting design. Vis. Eng..

[14] Iwaszczuk D., Stilla U. (2017). Camera pose refinement by matching uncertain 3D building models with thermal infrared image sequences for high quality texture extraction. ISPRS J. Photogramm. Remote Sens..

[15] Rea P., Ottaviano E. (2018). Design and development of an Inspection Robotic System for indoor applications. Robot. Comput.-Integr. Manuf..

[16] Merchán P., Adán A., Salamanca S., Domínguez V., Chacón R. (2012). Geometric and colour data fusion for outdoor 3D models. Sensors.

[17] Costanzo A., Minasi M., Casula G., Musacchio M., Buongiorno M.F. (2015). Combined use of terrestrial laser scanning and IR thermography applied to a historical building. Sensors.

[18] Wang X., Witz J.F., Bartali A.E., Jiang C. (2016). Infrared thermography coupled with digital image correlation in studying plastic deformation on the mesoscale level. Opt. Lasers Eng..

[19] Capozzoli L., Rizzo E. (2017). Combined NDT techniques in civil engineering applications: Laboratory and real test. Constr. Build. Mater..

[20] Yang M.D., Su T.C. (2009). Segmenting ideal morphologies of sewer pipe defects on CCTV images for automated diagnosis. Expert Syst. Appl..

[21] Yang M.D., Su T.C., Pan N.F., Yang Y.F. (2011). Systematic image quality assessment for sewer inspection. Expert Syst. Appl..

[22] Yang M.D., Su T.C., Pan N.F., Liu P. (2011). Feature extraction of sewer pipe defects using wavelet transform and co-occurrence matrix. Int. J. Wavel. Multiresolut. Inf. Process..

[23] Su T.C., Yang M.D., Wu T.C., Lin J.Y. (2011). Morphological segmentation based on edge detection for sewer pipe defects on CCTV images. Expert Syst. Appl..

[24] Lagüela S., Armesto J., Arias P., Herráez J. (2012). Automation of thermographic 3D modelling through image fusion and image matching techniques. Autom. Constr..

[25] Ham Y., Golparvar-Fard M. (2013). An automated vision-based method for rapid 3D energy performance modeling of existing buildings using thermal and digital imagery. Adv. Eng. Inform..

[26] Rangel J., Soldan S., Kroll A. 3D Thermal Imaging: Fusion of Thermography and Depth Cameras. Proceedings of the 12th International Conference on Quantitative InfraRed Thermography (QIRT).

[27] Adán A., Prado T., Prieto S.A., Quintana B. Fusion of Thermal Imagery and LiDAR Data for Generating TBIM Models. Proceedings of the 2017 IEEE Sensors.

[28] Schramm S., Rangel J., Kroll A. Data Fusion for 3D Thermal Imaging Using Depth and Stereo Camera for Robust Self-localization. Proceedings of the 2018 IEEE Sensors Applications Symposium (SAS).

[29] Kazi A.M., Ali M., Ayub K., Kalimuddin H., Zubair K., Kazi A.N., Artani A., Ali S.A. (2017). Geo-spatial reporting for monitoring of household immunization coverage through mobile phones: Findings from a feasibility study. Int. J. Med. Inform..

[30] Matarazzo T., Vazifeh M., Pakzad S., Santi P., Ratti C. (2017). Smartphone data streams for bridge health monitoring. Procedia Eng..

[31] Zhang H., Wei Q., Jiang Z. (2017). 3D reconstruction of space objects from multi-views by a visible sensor. Sensors.

[32] Lu Y., Wu Z., Chang R., Li Y. (2017). Building Information Modeling (BIM) for green buildings: A critical review and future directions. Autom. Constr..

[33] Lin Y.H., Tsai K.T., Lin M.D., Yang M.D. (2016). Design optimization of office building envelope configurations for energy conservation. Appl. Energy.

[34] Yang M.D., Lin M.D., Lin Y.H., Tsai K.T. (2017). Multiobjective optimization design of green building envelope material using a non-dominated sorting genetic algorithm. Appl. Therm. Eng..

[35] Rajeev P., Sanjayan J.G., Seenuth S.S. (2016). Assessment of thermal cracking in concrete roof tiles. Mater. Des..

[36] Li R., Zeng B., Liou M.L. (1994). A new three-step search algorithm for block motion estimation. IEEE Trans. Circuits Syst. Video Technol..

[37] Bracewell R. (1965). The Fourier Transform and Its Applications.

[38] Wolf P.R., Dewitt B.A. (2000). Elements of Photogrammetry with Applications in GIS.

[39] Yu H., Lin Z., Pan F. (2005). Applications and improve of H.264 in medical video compression. IEEE Trans. Circuits Syst. I.

[40] Lakamsani P., Zeng B., Liou M. An Enhanced Three Step Search Motion Estimation Method and Its VLSI Architecture. Proceedings of the IEEE International Symposium on Circuits and Systems, Circuits and Systems Connecting the World (ISCAS 96).

[41] VisualSFM: A Visual Structure from Motion System. http://ccwu.me/vsfm/.

[42] Open Multi-View Stereo Reconstruction Library. http://cdcseacave.github.io/openMVS/.

[43] Snavely N., Seitz S.M., Szeliski R. (2008). Modeling the world from internet photo collections. Int. J. Comput. Vis..

[44] Hartley R., Zisserman A. (2004). Multiple View Geometry in Computer Vision.

[45] Lourakis M.I.A., Argyros A.A. (2006). SBA: A software package for generic sparse bundle adjustment. ACM Trans. Math. Softw..

[46] Barnes C., Shechtman E., Finkelstein A., Goldman D.B. A Randomized Correspondence Algorithm for Structural Image Editing. Proceedings of the ACM SIGGRAPH 2009.

[47] Laurentini A. (1994). The visual hull concept for silhouette-based image understanding. IEEE Trans. Pattern Anal. Mach. Intell..

[48] Vu H.-H., Labatut P., Pons J.-P., Keriven R. (2012). High accuracy and visibility-consistent dense multiview stereo. IEEE Trans. Pattern Anal. Mach. Intell..

[49] Lillesand T.M., Kiefer R.W., Chipman J.W. (2008). Remote Sensing and Image Interpretation.

[50] Whelan T., Ma L., Bondarev E., de With P.H.N., McDonald J. (2015). Incremental and batch planar simplification of dense point cloud maps. Robot. Auton. Syst..

